# Low NDRG2 expression predicts poor prognosis in solid tumors: A meta-analysis of cohort study

**DOI:** 10.1097/MD.0000000000022678

**Published:** 2020-10-09

**Authors:** Aiqin Gu, Jie Xu, Jun Ye, Chuanmeng Zhang

**Affiliations:** a Nursing Department, Taizhou People's Hospital, Affiliated 5 to Nantong University; b The Center for Translational Medicine, Taizhou People's Hospital, Affiliated 5 to Nantong University, Taizhou, Jiangsu Province, China.

**Keywords:** NDRG2, solid tumor, prognosis, meta-analysis

## Abstract

**Background:**

As a member of the N-myc down-regulated gene family, N-Myc downstream-regulated gene 2 (NDRG2) contributes to the tumorigenesis of various types of cancers. However, the correlation between NDRG2 expression and the prognosis of solid tumor remains to be elucidated because of small sample sizes and inconsistent results in previous studies. In the present study, we conducted a systematic review and meta-analysis to explore the prognostic significance of NDRG2 in human solid tumors.

**Methods:**

PubMed, Web of Science, Embase, Chinese National Knowledge Infrastructure, and WanFang databases (up to April 2020) were searched for relevant studies that evaluated the impact of NDRG2 on clinical outcomes, including overall survival (OS), and disease-free survival (DFS), in solid tumors. Hazard ratios (HRs) with 95% confidence intervals (CIs) were pooled to assess the association between NDRG2 expression and the survival of patients with solid tumors. Odds ratios (ORs) with 95% CIs were pooled to estimate the correlation between NDRG2 expression and clinicopathologic characteristics in the patients.

**Results:**

A total of 13 eligible studies with 1980 patients were included in this meta-analysis. Low NDRG2 expression was significantly associated with poor OS (HR = 1.96, 95% CI: 1.60–2.40, *P* < .001) and DFS (HR = 2.70, 95% CI: 1.42–5.13, *P* = .002) in solid tumor. Furthermore, low NDRG2 expression was related to some phenotypes of tumor aggressiveness, such as clinical stage (OR = 3.21, 95% CI: 1.96–5.26, *P* < .001), lymph node metastasis (OR = 2.14, 95% CI: 1.49–3.07, *P* < .001), and degree of differentiation (OR = 0.60, 95% CI: 0.45–0.81, *P* = .001).

**Conclusions:**

NDRG2 may be a meaningful biomarker of poor prognosis and a potential therapeutic target for human solid tumors.

## 1 Introduction

Cancer's high morbidity and mortality make it a worldwide public health concern, and its mortality rate is higher than that of cardiovascular diseases in some countries.^[1,2]^ Despite rapid progress in targeted therapies and comprehensive treatments for cancers, the prognosis for most cancer patients is still poor.^[3]^ One of the greatest challenges in cancer treatment is the accurate prediction of the recurrence and outcome of each patient to determine which treatment strategies are appropriate. Although traditional pathological stages have been developed to predict clinical outcome and guide the treatment of patients with solid tumors, they fail to accurately predict prognosis. Thus, identification of more molecules involved in the occurrence and development of cancer might be of great significance in the recognition of potential markers and specific targets for cancer prevention and individualized treatment.^[4]^

N-Myc downstream regulatory gene 2 (NDRG2) belongs to the NDRG family, which is related to human cancer and nervous system diseases.^[5,6]^ NDRG2 is recently revealed to be a candidate tumor suppressor gene that plays an active role in controlling tumor growth and morbidity effect. Additionally, accumulative evidence indicated that overexpression of NDRG2 can significantly inhibit tumor growth, migration, proliferation, adhesion, and invasion.^[7,8]^ Moreover, NDRG2 is reportedly involved in the cellular metabolism processes, such as hormone, ionic, and liquid metabolism, as well as stress responses, such as responses to hypoxia and lipotoxicity.^[9–13]^ NDRG2 expression is decreased in various cancers, including lung,^[14]^ breast,^[15]^ colorectal,^[16]^ hepatocellular,^[17]^ pancreatic,^[18]^ and esophageal squamous cancers.^[19]^ Recent studies have demonstrated that low NDRG2 protein expression is closely related to poor prognosis of cancer patients.^[18,20–30]^ However, the prognostic impact of NDRG2 is inconclusive.^[26,31]^ Thus, we conducted this meta-analysis to evaluate the prognostic value of NDRG2 protein expression in various solid tumors.

## 2 Materials and methods

### 2.1 Ethics and dissemination

As the present meta-analysis was conducted based on previous published studies and did not involve direct contact with patients or alterations to patient care, ethical approval, and patient consent are not required.

### 2.2 Search strategy

We performed a comprehensive literature search using PubMed, Web of Science, EMBASE, Chinese National Knowledge Infrastructure, and WanFang databases (up to April 2020) for relevant studies that analyzed the prognostic value of NDRG2 in various cancers. The following search terms were used: (“N-Myc downstream-regulated gene 2” or “NDRG2”), and (“tumor” or “cancer” or “carcinoma” or “neoplasm”), and (“prognosis” or “outcome” or “survival”). We also manually filtered the reference lists of the searched articles to identify additional studies. Full-text articles published in English or Chinese were included.

### 2.3 Inclusion and exclusion criteria

Studies that were enrolled adhered to the following criteria:

1. human solid tumor was diagnosed by histopathology;
2. NDRG2 expression was measured in cancer tissues by immunohistochemistry (IHC) stain and divided into “high” and “low” or “positive” and ”negative” groups;
3. the relationship between NDRG2 expression and solid tumor prognosis, including overall survival (OS) or disease-free survival (DFS), was assessed; and
4. hazard ratios (HRs) with 95% confidence intervals (CIs) can be extracted directly or calculated with sufficient data.

Articles were excluded according to the following criteria:

1. reviews, letters, case reports, editorials, abstracts, expert opinions, or animal experiments;
2. miRNA expression was detected in tumor tissue;
3. studies without key information that could be used to estimate the HR and 95% CI;
4. patients were not divided into two groups based on NDRG2 expression; and
5. studies with sample sizes of less than 50.

### 2.4 Data extraction and quality assessment

Two investigators (GAQ and XJ) independently evaluated and extracted data from each study according to the predefined criteria. Any disagreements were resolved by reaching a consensus with a third investigator (ZCM). The following information was extracted from the eligible studies: first authors name, year of publication, country, cancer type, clinical stage, follow-up time, sample size, outcome endpoint, and HR estimation with 95% CI of low NDRG2 expression group versus high NDRG2 group. If univariate and multivariate HR estimations were both provided, then we preferred to use the latter to minimize bias.

The quality of all included studies was evaluated independently by 2 authors (GAQ and YJ) using the Newcastle-Ottawa Scale (NOS). Any discrepancies were resolved through discussion with another investigator (ZCM). The NOS score ranged from 0 to 9 based on the quality of selection, comparability, and outcome of interest. The investigations with scores higher than 6 had high-quality methodology.

### 2.5 Statistical analysis

Stata 12.0 (STATA Corp., College Station, TX) software was applied to perform all statistical analyses. The combined HRs and corresponding 95% CIs were calculated to evaluate the association of NDRG2 expression with patient survival. For the overall result, HR and 95% CI greater than 1 implied a worse prognosis in patients with low NDRG2 expression. Beyond that, the pooled odds ratio (OR) and their 95% CI were applied to assess the association between NDRG2 expression and the clinicopathological parameters of solid tumors. Heterogeneity among individual studies was evaluated by Chi-Squared Q and *I*-squared statistical tests. When the results (*I*^2^ > 50% or *P* < .05) indicated heterogeneity, the random-effects model was used for the meta-analysis. Otherwise, the fixed-effects model was adopted. Meta-regression and subgroup analyses were conducted to explore the sources of heterogeneity. Sensitivity analysis was performed to verify the outcome credibility by sequentially omitting each individual study. Publication bias was statistically evaluated using Begg and Egger tests and visually assessed with a funnel plot. In case of significant publication bias, the trim and fill method was applied to validate the robustness of the summary results.

## 3 Results

### 3.1 Search results and study characteristics

**Figure 1:**
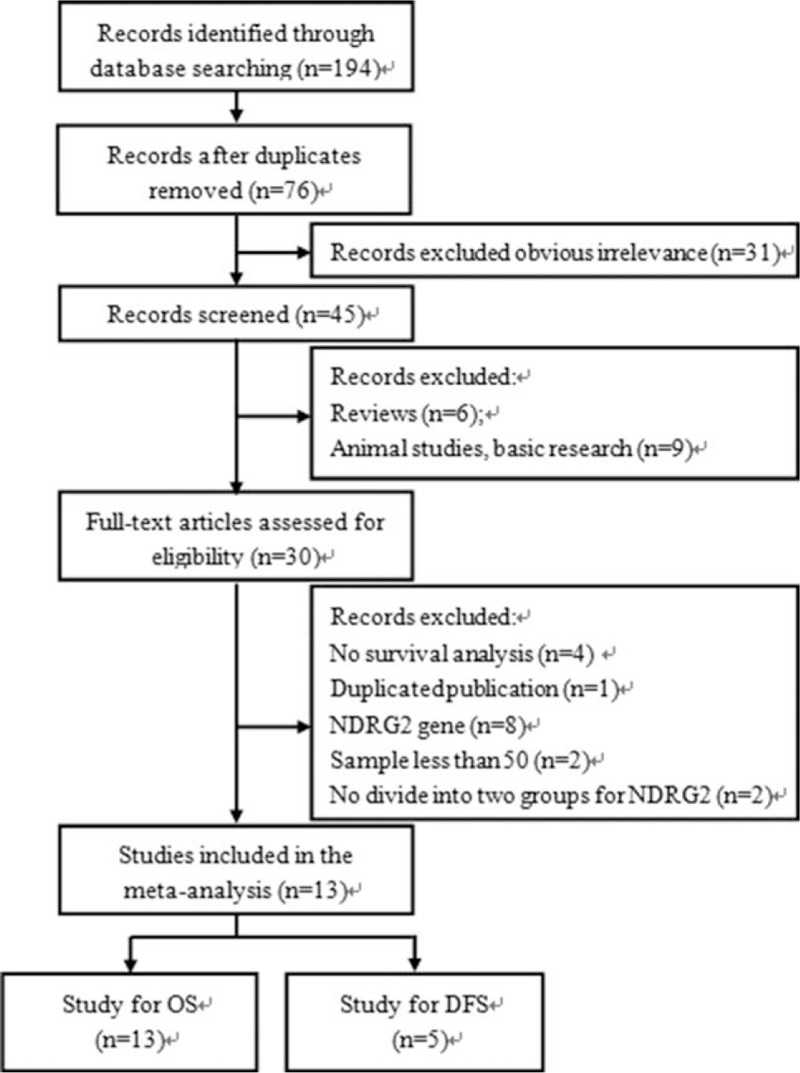
Flow diagram of the study selection process and specific reasons for exclusion in the meta-analysis.

According to the above-described search strategies, a total of 194 records were initially identified. After removing duplicate studies, 76 articles were required for further evaluation. After the exclusion of evidently irrelevant literature (n = 31), reviews (n = 6), and nonhuman studies (n = 9), 30 relevant full-text articles were assessed. Following the careful review of the full texts, 13 studies with 13 cohorts were finally identified as eligible and were then included in this meta-analysis. The process of literature selection is shown in Figure 1.

The included studies were published from 2012 to 2019 with a total of 1980 cancer patients from China,^[20–25,28–30]^ Korea,^[26,27,31]^ and Japan.^[18]^ Among all study cohorts, 2 evaluated colorectal cancer,^[20,26]^ breast cancer,^[24,31]^ and renal cell carcinoma,^[27,28]^ and single studies focused on hepatocellular carcinoma,^[21]^ cholangiocarcinoma,^[22]^ prostate cancer,^[25]^ gastric carcinomas,^[23]^ pancreatic cancer,^[18]^ lung cancer,^[30]^ and gallbladder carcinoma.^[29]^ The sample size ranged from 60 to 316. All included cohorts reported data on OS,^[18,20–31]^ and 5 of them including 919 patients also provided the data on DFS.^[24–27,31]^ The HR and 95% CI were directly obtained from 7 studies.^[20–22,25–27,31]^ Data from the remaining 6 studies were extracted using Kaplan–Meier survival curves.^[18,23,24,28–30]^ On the basis of the NOS, every cohort study was allocated a score of ≥6, suggesting that these studies were of high quality. Other characteristics of the included studies are described in detail in Table 1.

**Table 1:**
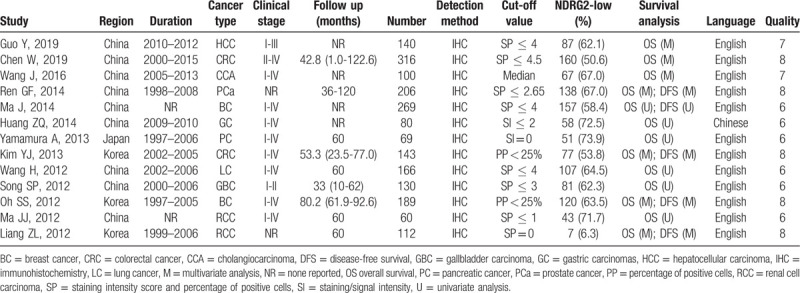
Main characteristics of the eligible studies.

### 3.2 Relationship between NDRG2 expression and prognosis

**Figure 2:**
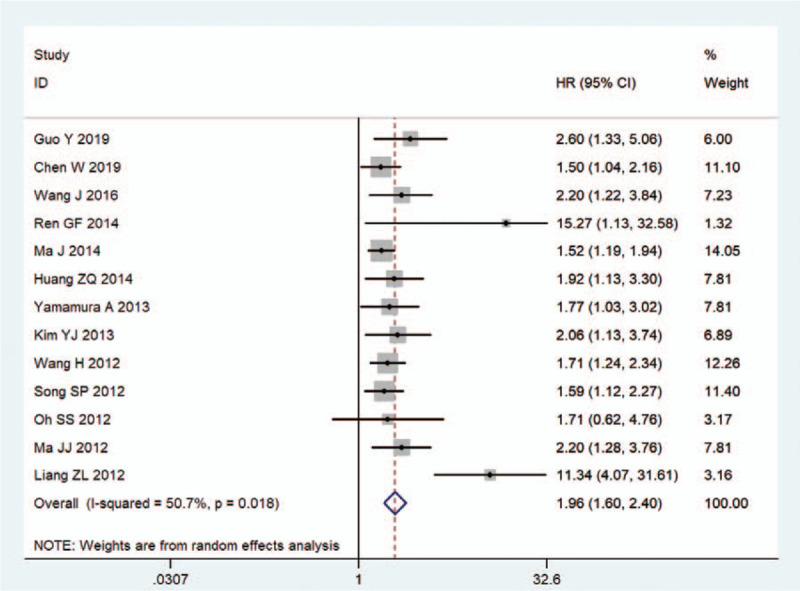
Forest plots of the overall outcomes for OS. The HRs for each trial are represented by the squares, and the horizontal lines crossing the square stand for the 95% CIs. The diamonds represent the estimated pooled effect of the overall outcome for OS in all solid tumors. All *P* values are two-sided.

All 13 included cohorts reported the results of OS toward NDRG2 expression with a total of 1980 cancer patients. Considering the significant heterogeneity among studies (*I*^2^ = 50.7%, *P* = .018), the random-effects model was applied to calculate the pooled HR and 95% CI (Table 2, Fig. 2). The results demonstrated that low expression level of NDRG2 was correlated with poor OS in human cancer (HR = 1.96, 95% CI: 1.60–2.40, *P* < .001, Table 2).

**Table 2:**
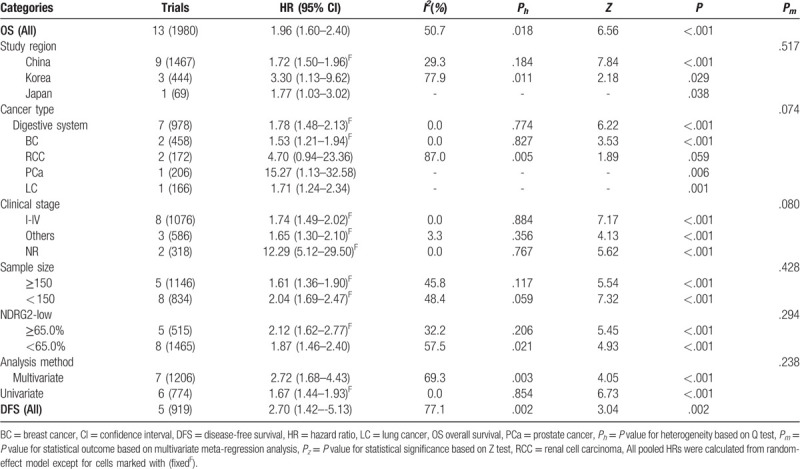
Summary of the meta-analysis results.

Considering the significant heterogeneity among studies, subgroup and meta-regression analyses were performed by focusing on the study region, cancer type, clinical stage, sample size, the proportion of patients with low NDRG2 expression, and analysis method to explore the sources of heterogeneity for OS (Table 2). In the subgroup analysis of study region, the low expression of NDRG2 was significantly associated with worse OS in China (HR = 1.72, 95% CI: 1.50–1.96, *P* < .001), Korea (HR = 3.30, 95% CI: 1.13–9.62, *P* = .029), and Japan (HR = 1.77, 95% CI: 1.03–3.02, *P* = .038). Regarding cancer type subgroup analysis, the overall results showed that the negative expression of NDRG2 had an unfavorable impact on OS in patients with digestive system malignancies (HR = 1.78, 95% CI: 1.48–2.13, *P* < .001), breast cancer (HR = 1.53, 95% CI: 1.21–1.94, *P* < .001), prostate cancer (HR = 15.27, 95% CI: 1.13–32.58, *P* = .006), and lung cancer(HR = 1.71, 95% CI: 1.24–2.34, *P* = .001), but such association was not observed for patients with renal cell carcinoma (HR = 4.70, 95% CI: 0.94–23.36, *P* = .059). Regarding clinical stage subgroup analysis, a significant relationship was found between NDRG2 expression and prognosis in patients with cancer stages I-IV (HR = 1.74, 95% CI: 1.49–2.02, *P* < .001), others (HR = 1.65, 95% CI: 1.30–2.10, *P* < .001), and NR (HR = 12.29, 95% CI: 5.12–29.50, *P* < .001). When subgroup analysis was performed based on sample size, low NDRG2 expression was significantly correlated with short OS in both small (HR = 1.61, 95% CI: 1.36–1.90, *P* < .001) and large (HR = 2.04, 95% CI: 1.69–2.47, *P* < .001) sample sizes. Additionally, a subgroup analysis of OS was performed according to the proportion of patients with low NDRG2 expression, as determined by the cut-off value. The results showed that low NDRG2 protein expression was associated with poor OS in high (HR = 2.12, 95% CI: 1.62–2.77, *P* < .001) and low (HR = 1.87, 95% CI: 1.46–-2.40, *P* < .001) proportion groups. Similarly, patients with low NDRG2 expression had worse OS than those with high expression in multivariate (HR = 2.72, 95% CI: 1.68–4.43, *P* < .001) and univariate (HR = 1.67, 95% CI: 1.44–1.93, *P* < .001) analyses. Furthermore, meta-regression demonstrated that study region (*P* = .517), cancer type (*P* = .074), clinical stage (*P* = .080), sample size (*P* = .428), the proportion of patients with low NDRG2 expression (*P* = .294), and analysis method (*P* = .238) were not able to explain the source of heterogeneity.

**Figure 3:**
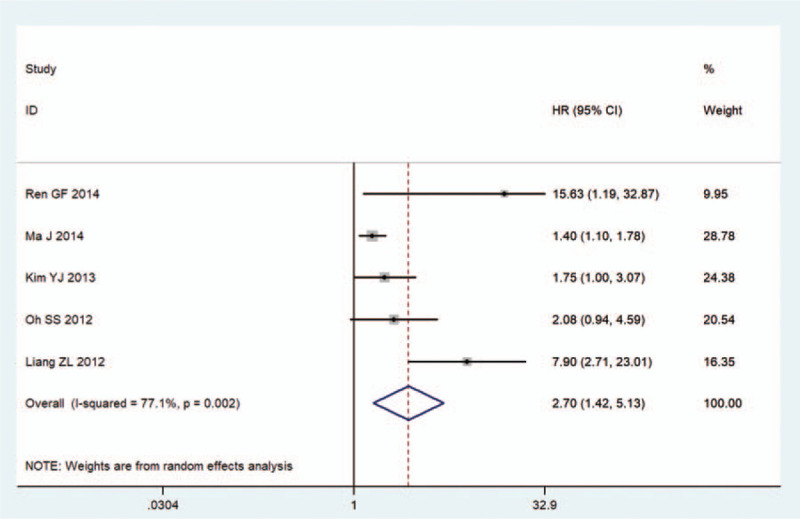
Forest plots of the overall outcomes for DFS. The HRs for each trial are represented by the squares, and the horizontal lines crossing the square stand for the 95% CIs. The diamonds represent the estimated pooled effect of the overall outcome for DFS in all solid tumors. All *P* values are two-sided.

Five cohorts comprising 919 participants reported the primary endpoint of DFS. Considering the significant heterogeneity (*I*^2^ = 77.1%, *P* = .002), the random-effects model was applied. The pooled HR was 2.70 (95% CI: 1.42–5.13, *P* = .002; Table 2, Fig. 3), demonstrating that low NDRG2 expression predicted reduced DFS.

### 3.3 Sensitivity analysis and publication bias

**Figure 4:**
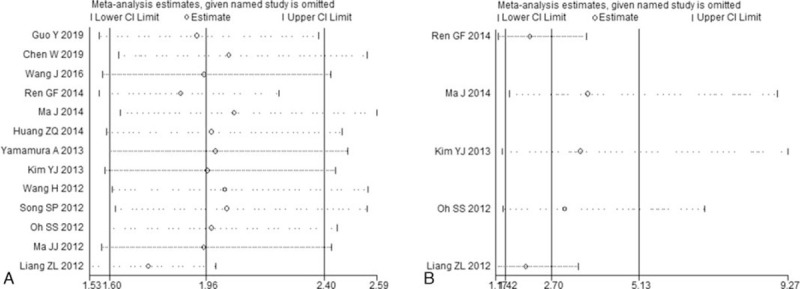
Effects of individual studies on pooled HRs for NDRG2 and survival in solid tumors. (A) Result of sensitivity analysis for pooled OS estimation. (B) Result of sensitivity analysis for pooled DFS estimation.

Sensitivity analysis results suggested that the pooled HR estimations for OS (Fig. 4A) or DFS (Fig. 4B) were not influenced by the combined overall results after the sequential omission of each individual study, thereby indicating that the results were stable and reliable.

**Figure 5:**
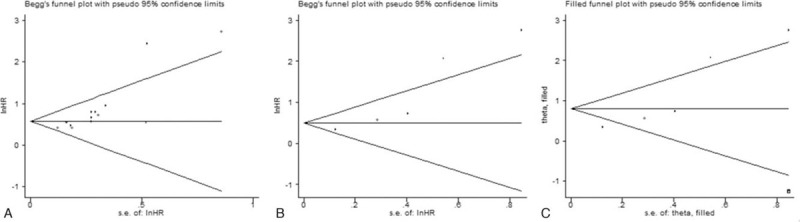
Beggs funnel plots for assessing potential publication bias in studies of NDRG2 in patients with solid tumors. Each study represented by one circle. The horizontal line represented the pooled effect estimate. (A) Funnel plot of publication bias for OS. (B) Funnel plot of publication bias for DFS. (C) Funnel plot adjusted with trim and fill methods for DFS.

The shapes of the funnel plot showed a certain degree of apparent asymmetry for OS (Fig. 5A), as confirmed by Beggs (*P* = .003) and Eggers (*P* = .001) tests. However, the trim and fill analysis (no new studies added) did not show any indication of publication bias, which suggested that the results were robust and reliable. A significant publication bias was found by Eggers test concerning the pooled result of DFS (*P* = .027), but this was not found by Beggs test (*P* = .086), as depicted by the funnel plot shape (Fig. 5B). After conducting the trim-and-fill analysis, 1 non-published study was needed to balance the funnel plot (Fig. 5C), and the adjusted HR and 95% CI slightly changed but remained significant (HR = 2.22, 95% CI: 1.17–4.25, *P* = .015), indicating that potential publication bias had minimal impact on the overall results.

### 3.4 Association between NDRG2 expression and clinicopathological features.

To further explore the prognostic value of NDRG2 in solid tumors, the combined results of the correlations were identified between NDRG2 expression and the clinicopathological features of patients with solid tumors (Table 3). Low NDRG2 expression was related to some phenotypes of tumor aggressiveness, such as advanced clinical stage (OR = 3.21, 95% CI: 1.96–5.26, *P* < .001), positive lymph node metastasis (OR = 2.14, 95% CI: 1.49–3.07, *P* < .001), and poor degree of differentiation (OR = 0.60, 95% CI: 0.45–0.81, *P* = .001). However, no significant relationship was found between NDRG2 expression and other clinicopathological features, such as age, gender, and tumor size.

**Table 3:**
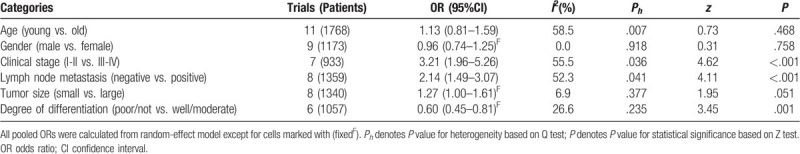
Meta-analysis of NDRG2 and clinicopathological features in cancer patients.

## 4 Discussion

Decreased NDRG2 expression is demonstrated in multiple solid tumors and the expression level could serve as a potential prognostic marker for OS and DFS. However, the relationship between NDRG2 expression and its prognosis remains controversial. In this study, results of the meta-analysis demonstrated that downregulation of NDRG2 was associated with poor OS (HR = 1.96, 95% CI: 1.60–2.40, *P* < .001) and DFS (HR = 2.70, 95% CI: 1.42–5.13, *P* = .002) in various types of cancers. Considering the heterogeneity between studies, subgroup and mete-regression analyses were conducted by focusing on study region, cancer type, clinical stage, sample size, the proportion of patients with low NDRG2 expression, and analysis method to explore sources of heterogeneity for OS. However, these factors did not explain the source of heterogeneity. After careful analysis, we found that the heterogeneity mainly comes from Ren GFs article and Liang ZLs research. The reasons for the difference between these 2 studies and other studies are the small sample sizes and the small numbers of patients with low NDRG2 expression. Moreover, sensitivity analysis and publication bias demonstrated that the results were stable and reliable. Meanwhile, the association between NDRG2 expression and different clinicopathological features was consistent. Therefore, our meta-analysis showed that decreased NDRG2 expression was statistically correlated with poor prognosis in cancer patients.

A better understanding of the molecular mechanisms underlying the function of NDRG2 in tumorigenesis and tumor progression helps us elucidate prognostic results.

First, differentiation is important in solid tumors; undifferentiated histology is often a marker of tumor aggressiveness and poor prognosis.^[5]^ One characteristic of cell differentiation is the reduced rate of cell cycle progression. The G1-S transition is driven by cyclin-dependent kinase (CDK) 2, which is controlled by CDK inhibitor p21^WAF1^ (p21) and p27^KIP1^ (p27).^[32]^ The stability of p21 and p27 in cells is primary regulated by S-phase kinase-associated protein 2 (Skp2).^[33]^ The induction of NDRG2 increases Skp2 expression by promoting β-catenin nuclear translocation, and consequently accelerates the ubiquitination and degradation of p21 and p27, thereby inhibiting cell differentiation.^[5]^ On the other hand, cyclin D1 belongs to a highly conserved cyclin family, and its members are characterized by dramatic periodic changes in protein abundance during the cell cycle.^[34]^ High cyclin D1 expression changes the cell cycle process and possibly contributes to tumorigenesis.^[13,34]^ Induction of NDRG2 reduces c-Jun phosphorylation at Ser63, which is followed by the attenuation of the transcriptional activator protein-1. This further down-regulates cyclin D1, thereby causing the cell cycle arrest at G1/S.^[35]^ Other proteins and pathways are also involved in proliferation, such as P38 mitogen-activated protein kinase.^[36]^

Second, migration and invasion of cancer cells to the surrounding tissues and vasculature are important initial steps in cancer metastasis, which is the main cause of cancer-related death.^[37]^ In esophageal cancer, the overexpression of NDRG2 has been shown to inhibit tumor migration, invasion, and epithelial-mesenchymal transition by inhibiting the protein kinase B/X-linked inhibitor of apoptosis protein signaling pathway.^[38]^ Moreover, NDRG2 contributes to the migration and invasion of oral squamous cell carcinoma and breast cancer through the inhibition of the phosphatidylinositol 3-kinase/protein kinase B signaling pathway.^[39,40]^ In addition, NDRG2 reportedly promotes cancer cell migration and invasion by upregulating the expressions of β-catenin, matrix metalloproteinase (MMP)-2, and MMP-9 and by decreasing the expression of E-cadherin.^[26,41,42]^

In addition to malignant growth, proliferation, and invasion, metabolic abnormality is currently regarded as a new malignant phenotype of cancer cells.^[43]^ Tumor cells use glucose and glutamine as their main energy sources and precursor intermediates; thus enhanced glycolysis and glutamine dissolution are the major hallmarks of tumor metabolic reprogramming.^[42]^ In colorectal cancer cells, NDRG2 inhibits glucose consumption and production, as well as glutamine consumption and glutamate production, by repressing c-Myc expression, and the transporters and catalytic enzymes involved include glucose transporter 1, hexokinase 2, pyruvate kinase M2 isoform, lactate dehydrogenase A, glutamine transporter ASC amino acid transporter 2, and glutaminase 1.^[42]^

However, several limitations also exist in this study. First, the patients included in this meta-analysis study were all Asian, which may affect the applicability of our results. Secondly, the studies included in the meta-analysis were all retrospective works; studies with positive results were more likely to be published than negative results. Furthermore, although all eligible cohorts detected the NDRG2 expression by IHC, the cutoff value varied across different studies, which might have caused bias in the pooled analysis. Although we did not restrict the language of the literature, only studies published in English and Chinese were included in the meta-analysis

In summary, our meta-analysis demonstrated that low NDRG2 expression is related to unfavorable outcomes, including OS and DFS, in patients with solid tumors. Thus, NDRG2 may be potentially used as a cancer therapy target.

## Author contributions

**Conceptualization:** Chuanmeng Zhang.

**Data analysis:** Aiqin Gu, Jie Xu, Jun Ye, Chuanmeng Zhang.

**Original draft writing:** Aiqin Gu, Jie Xu.

**Review & editing:** Chuanmeng Zhang, Jun Ye.

## Abbreviations

Abbreviations: CI = confidence interval, DFS = disease-free survival, HR = Hazard ratio, MMP = matrix metalloproteinase, NDRG2 = N-Myc downstream-regulated gene 2, NOS = Newcastle-Ottawa Scale, NR = none reported, OR = odds ratio, OS = overall survival.

## References

[1] Torre LA Bray F Siegel RL. Global cancer statistics, 2012. CA 2015;65:87–108. 25651787 10.3322/caac.21262

[2] Wang S Chen B Zhu Z. CDC20 overexpression leads to poor prognosis in solid tumors. Medicine 2018;97:e13832. 30593179 10.1097/MD.0000000000013832 PMC6314760

[3] Yu M Hao B Zhan Y. Krüppel-like factor 4 expression in solid tumor prognosis: A meta-analysis. Clinica Chimica Acta 2018;485:50–9. 10.1016/j.cca.2018.06.030 29940144

[4] Wen Y Shu F Chen Y. The prognostic value of HOXA13 in solid tumors: a meta-analysis. Clinica Chimica Acta 2018;483:64–8. 10.1016/j.cca.2018.04.024 29678634

[5] Shen L Qu X Li H. NDRG2 facilitates colorectal cancer differentiation through the regulation of Skp2-p21/p27 axis. Oncogene 2018;37:1759–74. 29343851 10.1038/s41388-017-0118-7 PMC5874257

[6] Rong X Sun Y Liu D. The pathological roles of NDRG2 in Alzheimer's disease, a study using animal models and APPwt-overexpressed cells. CNS Neurosci Therapeut 2017;23:667–79. 10.1111/cns.12716 PMC6492714 28670853

[7] Fu Q Gao Y Yang F. Suppression of microRNA-454 impedes the proliferation and invasion of prostate cancer cells by promoting N-myc downstream-regulated gene 2 and inhibiting WNT/β-catenin signaling. Biomed Pharmacother 2018;97:120–7. 29080452 10.1016/j.biopha.2017.10.115

[8] Hu W Yang Y Fan C. Clinical and pathological significance of N-Myc downstream-regulated gene 2 (NDRG2) in diverse human cancers. Apoptosis 2016;21:675–82. 27113371 10.1007/s10495-016-1244-3

[9] Li Y Liu C Hou W. Retrograde ductal administration of the adenovirus-mediated NDRG2 gene leads to improved sialaden hypofunction in estrogen-deficient rats. Mol Ther 2014;22:908–18. 24343104 10.1038/mt.2013.286 PMC4015234

[10] Li Y Yang J Li S. N-myc Downstream-regulated Gene 2, a novel estrogen-targeted gene, is involved in the regulation of Na+ /K+-ATPase. J Biol Chem 2011;286:32289–99. 21771789 10.1074/jbc.M111.247825 PMC3173200

[11] Liu J Zhang J Wang X. HIF-1 and NDRG2 contribute to hypoxia-induced radioresistance of cervical cancer Hela cells. Exp Cell Res 2010;316:1985–93. 20206160 10.1016/j.yexcr.2010.02.028

[12] Shen L Liu X Hou W. NDRG2 is highly expressed in pancreatic β cells and involved in protection against lipotoxicity. Cell Molec Life Sci 2010;67:1371–81. 20127388 10.1007/s00018-010-0258-1 PMC11115835

[13] Hu W Fan C Jiang P. Emerging role of N-myc downstream-regulated gene 2 (NDRG2) in cancer. Oncotarget 2016;7:209–23. 26506239 10.18632/oncotarget.6228 PMC4807993

[14] Li S Wang W Li B. Expression of NDRG2 in human lung cancer and its correlation with prognosis. Med Oncol 2013;30:421. 23307246 10.1007/s12032-012-0421-7 PMC3586402

[15] Liu N Wang L Liu X. Promoter methylation, mutation, and genomic deletion are involved in the decreased NDRG2 expression levels in several cancer cell lines. Biochem Biophys Res Commun 2007;358:164–9. 17470364 10.1016/j.bbrc.2007.04.089

[16] Feng L Xie Y Zhang H. Down-regulation of NDRG2 gene expression in human colorectal cancer involves promoter methylation and microRNA-650. Biochem Biophys Res Commun 2011;406:534–8. 21352815 10.1016/j.bbrc.2011.02.081

[17] Godeke J Luxenburger E Trippel F. Low expression of N-myc downstream-regulated gene 2 (NDRG2) correlates with poor prognosis in hepatoblastoma. Hepatol Int 2016;10:370–86. 26646663 10.1007/s12072-015-9686-1

[18] Yamamura A Miura K Karasawa H. Suppressed expression of NDRG2 correlates with poor prognosis in pancreatic cancer. Biochem Biophys Res Commun 2013;441:102–7. 24134849 10.1016/j.bbrc.2013.10.010

[19] Shi H Li N Li S. Expression of NDRG2 in esophageal squamous cell carcinoma. Cancer Sci 2010;101:1292–9. 20331630 10.1111/j.1349-7006.2010.01529.x PMC11158127

[20] Chen W Peng J Ou Q. Expression of NDRG2 in human colorectal cancer and its association with prognosis. J Cancer 2019;10:3373–80. 31293640 10.7150/jca.31382 PMC6603412

[21] Guo Y Li X Sun X. Combined aberrant expression of NDRG2 and LDHA Predicts hepatocellular carcinoma prognosis and mediates the anti-tumor effect of gemcitabine. Int J Biol Sci 2019;15:1771–86. 31523182 10.7150/ijbs.35094 PMC6743297

[22] Wang J Xie C Pan S. N-myc downstream-regulated gene 2 inhibits human cholangiocarcinoma progression and is regulated by leukemia inhibitory factor/MicroRNA-181c negative feedback pathway. Hepatology 2016;64:1606–22. 27533020 10.1002/hep.28781

[23] Huang ZQ Liu S Qian G. Study on expression of NDRG2 protein in gastric cancer tissue. J Clin Exp Med 2014;13:1954–7.

[24] Ma J Liu W Guo H. N-myc downstream-regulated gene 2 expression is associated with glucose transport and correlated with prognosis in breast carcinoma. Breast Cancer Res 2014;16:R27. 24636131 10.1186/bcr3628 PMC4053222

[25] Ren GF Tang L Yang AQ. Prognostic impact of NDRG2 and NDRG3 in prostate cancer patients undergoing radical prostatectomy. Histol Histopathol 2014;29:535–42. 24222185 10.14670/HH-29.10.535

[26] Kim YJ Kang HB Yim HS. NDRG2 positively regulates E-cadherin expression and prolongs overall survival in colon cancer patients. Oncol Rep 2013;30:1890–8. 23900729 10.3892/or.2013.2642

[27] Liang ZL Kang K Yoon S. NDRG2 is involved in the oncogenic properties of renal cell carcinoma and its loss is a novel independent poor prognostic factor after nephrectomy. Ann Surg Oncol 2012;19:2763–72. 22246425 10.1245/s10434-011-2204-3

[28] Ma J Kong L Liao C. Suppression of MMP-9 activity by NDRG2 expression inhibits clear cell renal cell carcinoma invasion. Med Oncol 2012;29:3306–13. 22692967 10.1007/s12032-012-0265-1

[29] Song S Zhang S Liu R. NDRG2 down-regulation and CD24 up-regulation promote tumor aggravation and poor survival in patients with gallbladder carcinoma. Med Oncol 2012;29:1879–85. 22135002 10.1007/s12032-011-0110-y

[30] Wang H Wang W Wang X. Reduced N-Myc downstream-regulated gene 2 expression is associated with CD24 upregulation and poor prognosis in patients with lung adenocarcinoma. Med Oncol 2012;29:3162–8. 22528516 10.1007/s12032-012-0231-y

[31] Oh S Kim D Kim D. NDRG2 correlated with favorable recurrence-free survival inhibits metastasis of mouse breast cancer cells via attenuation of active TGF-β production. Carcinogenesis 2012;33:1882–8. 22696597 10.1093/carcin/bgs211

[32] Pfeuty B David-Pfeuty T Kaneko K. Underlying principles of cell fate determination during G1 phase of the mammalian cell cycle. Cell Cycle 2008;7:3246–57. 18843205 10.4161/cc.7.20.6853

[33] Kim S Lee H Gu Kang H. Ablation of galectin-3 induces p27KIP1-dependent premature senescence without oncogenic stress. Cell Death Diff 2014;21:1769–79. 10.1038/cdd.2014.88 PMC4211374 24971481

[34] Musgrove EA Caldon CE Barraclough J. Cyclin D as a therapeutic target in cancer. Nat Rev Cancer 2011;11:558–72. 21734724 10.1038/nrc3090

[35] Kim YJ Yoon SY Kim J. NDRG2 suppresses cell proliferation through down-regulation of AP-1 activity in human colon carcinoma cells. Int J Cancer 2009;124:7–15. 18844221 10.1002/ijc.23945

[36] Yong HY Koh MS Moon A. The p38 MAPK inhibitors for the treatment of inflammatory diseases and cancer. Expert Opin Investing Drugs 2009;18:1893–905. 10.1517/13543780903321490 19852565

[37] Duff D Long A. Roles for RACK1 in cancer cell migration and invasion. Cellular Signalling 2017;35:250–5. 28336233 10.1016/j.cellsig.2017.03.005

[38] Yang C Zheng X Ye K. NDRG2 suppresses proliferation, migration, invasion and epithelial-mesenchymal transition of esophageal cancer cells through regulating the AKT/XIAP signaling pathway. Int J Biochem Cell Biol 2018;99:43–51. 29530788 10.1016/j.biocel.2018.03.003

[39] Furuta H Kondo Y Nakahata S. NDRG2 is a candidate tumor-suppressor for oral squamous-cell carcinoma 2010;391ss:1785–91. 10.1016/j.bbrc.2009.12.156 20045673

[40] Park Y Shon S Kim A. SOCS1 induced by NDRG2 expression negatively regulates STAT3 activation in breast cancer cells. Biochem Biophys Res Commun 2007;363:361–7. 17888401 10.1016/j.bbrc.2007.08.195

[41] Shon S Kim A Kim JY. Bone morphogenetic protein-4 induced by NDRG2 expression inhibits MMP-9 activity in breast cancer cells. Biochem Biophys Res Commun 2009;385:198–203. 19450561 10.1016/j.bbrc.2009.05.038

[42] Xu X Li J Sun X. Tumor suppressor NDRG2 inhibits glycolysis and glutaminolysis in colorectal cancer cells by repressing c-Myc expression. Oncotarget 2015;6:26161–76. 26317652 10.18632/oncotarget.4544 PMC4694893

[43] Hanahan D Weinberg RA. Hallmarks of cancer: the next generation. cell 2011;144:646–74. 21376230 10.1016/j.cell.2011.02.013

